# Chirality and morphology-dependent siRNA delivery for targeted lung cancer therapy with intrinsically chiral gold nanoparticles

**DOI:** 10.1016/j.mtbio.2026.103745

**Published:** 2026-10-09

**Authors:** Qiusha Li, Jinglei Xu, En Zhang, Chen Liu, Wuyi Liu, Kaichuan Yan, Doudou Xu, Jian Wang

**Affiliations:** a Key Laboratory of Biomedical Analytics (Southwest University), Chongqing Science and Technology Bureau, College of Pharmaceutical Sciences, Southwest University, Chongqing, 400715, PR China; b Chongqing Key Laboratory of Quality Control and Safety Evaluation of Active Pharmaceutical Ingredients, Chongqing Institute for Food and Drug Control, Chongqing, 401121, PR China; c Chongqing Pharmaceutical Science Technology School, Chongqing, 400700, PR China

**Keywords:** RNA interference, Nanodelivery systems, Intrinsic chirality, PD-L1, Lung cancer

## Abstract

The clinical translation of RNA interference (RNAi) for lung cancer treatment remains hampered by the lack of delivery vectors that concurrently optimize cellular uptake, target specificity, and in vivo stability. This work reports an intrinsically chiral gold nanoparticle (Au NP) platform with precisely controlled morphology for targeted siRNA delivery. Through a full 2 × 2 factorial design (2 chiralities × 2 morphologies), the work decouples the individual and combined contributions of these two parameters. It is found that right-handed chiral nanoparticles (D-NPs) show significantly faster cellular internalization and higher gene silencing potency than left-handed (L-NPs) and achiral (DL-NPs) counterparts. Beyond chirality, nanoparticle morphology serves as an equally critical parameter: the rhombicuboctahedral (RCO) architecture yields markedly higher silencing efficacy than cubic counterparts with D-chirality. Furthermore, proteomic analysis revealed that Au RCO nanoparticles with different chirality displayed distinct functional enrichment profiles. By D-RCO combination with minimal surface modification, siRNA targeting PD-L1 achieves potent knockdown and robust inhibition of H460 lung cancer proliferation both in vitro and in vivo. This work establishes chirality and morphology as co-designable parameters for gene delivery and provides a mechanistic blueprint for translating chiral nanomaterials into solid tumor therapy.

## Graphical abstract

This work establishes intrinsically chiral Au RCO as a versatile platform for targeted siRNA immunotherapy in lung cancer. Mechanistic studies reveal that it exhibits pronounced chirality- and morphology-dependent properties, enabling efficient gene delivery and targeted gene silencing. The optimally designed D-Au RCO@mPEG@siPD-L1 nanoformulation achieves robust PD-L1 knockdown and exerts potent antitumor activity against PD-L1-high H460 cells in a T cell-independent manner.

**Figure:**
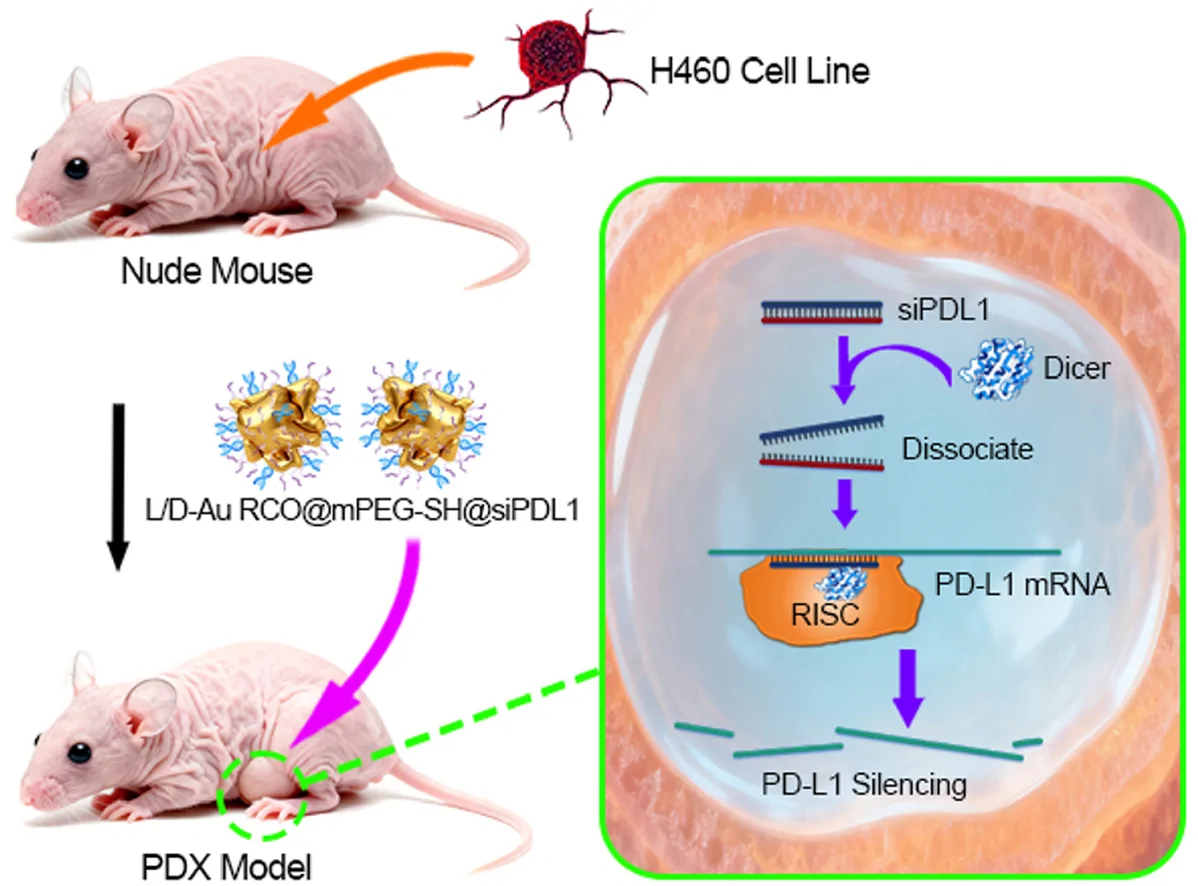

## 1 Introduction

RNA interference (RNAi) has been revitalized as a therapeutic modality following the FDA approvals of three siRNA drugs, including Onpattro, Givlaari, and Oxlumo, between 2018 and 2020 [1]. Despite its immense potential in oncology, the clinical translation of siRNA-based cancer therapy remains bottlenecked by several intertwined barriers: poor cellular uptake, susceptibility to nuclease degradation in vivo, and off-target effects [2], [3], [4]. Therefore, the development of delivery systems capable of efficiently encapsulating siRNA, protecting it from enzymatic degradation, and facilitating its functional intracellular delivery is essential for clinical translation.

Nanomaterials have been widely adopted for drug delivery owing to their excellent physicochemical properties. Various nanocarriers, including hydrogels [5], liposomes [6], metal-organic frameworks [7], [8], and gold nanoclusters [9], [10], have been engineered via chemical conjugation and surface cross-linking to achieve precise intracellular and in vivo delivery [11], [12]. Among these, gold nanoparticles (Au NPs) are particularly attractive due to their biocompatibility, ease of surface functionalization, and tunable photothermal properties, which enable controlled drug release and combination therapies [13], [14]. Conjugation with targeting ligands further allows active targeting and theranostic applications [15], [16]. Beyond biocompatibility and loading capacity, stereospecific interactions between nanocarriers and membrane proteins strongly influence delivery efficiency. Numerous studies have modified achiral nanomaterials with chiral ligands to enable specific recognition of chiral biomolecules and enhance cellular uptake [17], [18], [19]. However, these surface modification strategies suffer from inherent drawbacks, including immunogenicity and toxicity risks associated with ligand detachment in vivo [20], [21].

Recently, intrinsically chiral Au NPs have attracted considerable interest in biomedicine, with applications in therapy [22], [23], [24], photocatalysis [25], and chiral drug resolution [26]. Unlike surface-modified chiral particles, intrinsically chiral Au NPs prepared via seeded growth exhibit stable chiral structures that are resistant to ligand desorption [27]. They can be precisely synthesized with tunable morphologies and strong chiroptical signals [28], [29], [30], making them ideal platforms for biosensing [31], [32], immunotherapy [33], [34], and targeted delivery [35]. Despite these advances, several critical gaps remain regarding their application in siRNA delivery. First, while chirality has been implicated in modulating cellular uptake, the molecular mechanism by which intrinsic chirality governs membrane protein interactions and subsequent intracellular trafficking remains largely unknown. Second, it is unclear whether chirality influences not only the quantity but also the quality of cellular uptake. Specifically, whether it selectively engages pro-endocytic membrane proteins while suppressing negative regulatory pathways. Third, the role of nanoparticle morphology in this process has been largely treated as secondary, with little understanding of whether and how it interacts with chirality to govern overall delivery performance.

Herein, these gaps is addressed by designing a stereoselective chiral nanoplatform with intrinsically chiral Au NPs as siRNA carriers. Two distinct crystal morphologies are prepared with rhombi cuboctahedra (RCO) and cubes morphologies and generated right-handed (D-) and left-handed (L-) intrinsically chirality via secondary growth [36]. Through a complete 2 × 2 factorial design (D/L × RCO/Cube), ‌the work systematically decouples‌ the individual and combined contributions of chirality and morphology to siRNA delivery. Beyond physical characterization (Zeta potential, CD spectroscopy, SEM) and functional evaluation (GFP silencing), ‌it employs‌ high-throughput proteomic profiling to reveal the chirality-associated differential protein expression profiles. Quantitative binding assays (microscale thermophoresis, MST) further confirm that D-Au RCO exhibits ∼1.4-fold siRNA affinity compared to L-Au RCO. Beyond chirality, ‌the work identifies‌ morphology as a comparably critical design parameter: the RCO architecture consistently outperforms its cubic counterparts in promoting cellular binding and boosting intracellular siRNA bioavailability. This dual optimization of chirality and morphology culminates in the D-RCO configuration serving as the most potent siRNA delivery platform.

Based on this optimized D-RCO configuration, ‌the work fabricates‌ the Au RCO@mPEG-SH@siPD-L1 nanoplatform to achieve targeted silencing of PD-L1 expression [37] and downregulate STAT3 signaling [38], thereby inhibiting H460 lung cancer cell proliferation [39] while potentially modulating the immune microenvironment in vivo. Notably, by co-opting chirality and morphology, our platform achieves potent tumor inhibition without complex, detachable targeting ligands, substantially mitigating the translational risks associated with surface-coating instability. Compared with conventional vectors, this intrinsically chiral Au NP-based system overcomes the limitations of low uptake and poor targeting through chirality-morphology synergy, enabling precise siRNA delivery and effective tumor inhibition. This work provides a molecularly-grounded mechanistic blueprint for chirality- and morphology-optimized nanocarriers, expands the chiral nanomedicine toolbox, and offers a new paradigm for advanced gene delivery systems toward solid tumor therapy.

## 2 Material and methods

### 2.1 Materials

Tetrachloroauric acid (HAuCl_4_·3H_2_O), potassium iodide (KI) and sodium chloride (NaCl) were purchased from Macklin Reagent (Shanghai, China). L-cysteine (L-Cys), D-cysteine (D-Cys), L-ascorbic acid (L-AA) and sodium borohydride (NaBH_4_) were purchased from Aladdin Reagent Co., Ltd. (Shanghai, China). Cetyltrimethylammonium bromide (CTAB) and cetyltrimethylammonium chloride (CTAC) were obtained from Genview Reagent Co., Ltd. (Beijing, China). Cell counting kit-8 (CCK-8), Annexin V-FITC apoptosis detection Kit, immunofluorescence staining and reverse transcription -quantitative PCR (RT-qPCR)- related reagents were acquired from Accurate Biology Co. Ltd. (Changsha, China). All other chemicals were of analytical grade without further purification and prepared with deionized water (18 MΩ cm). All siRNAs (Table S1) and primers (Table S2) were purchased from Sangon Biotech Co. Ltd. (Shanghai, China).

### 2.2 Synthesis of chiral AuRCO and cube

For the synthesis of Chiral Au NPs: First, the seed solution was prepared by adding 3.95 mL of water, 10 μL of 100 mM chloroauric acid, 800 μL of 100 mM CTAB, 240 μL of 100 mM L-AA and 10 μL of 100 μM L/D-Cys in sequence and mixing thoroughly. Secondly, 20 μL of single-crystalline gold RCO and gold cube were added to the growth solution, mixed well and placed in a 30 °C water bath for 1.5 h. Finally, the synthesized chiral Au NPs were centrifuged at 3500 rpm for 4 min. After being washed twice with water, they were stored in 1 mL of 1 mM CTAB aqueous solution for subsequent surface modification and application.

### 2.3 Cell culture

H460 and A549 cells were obtained from Baidi Biotech. GFP-A549 cells were kindly provided by Prof. Yanlan Liu (Hunan University). All cells were cultured in DMEM containing 1.0 mg/mL D-glucose, 10% FBS, and 1% penicillin-streptomycin at 37 °C in a humidified 5% CO_2_ atmosphere.

### 2.4 Gene silencing effect evaluation

The gene knockdown efficacy of DL-/L-/D-Au NPs@siRNA targeting green fluorescent protein (GFP) was assessed. In vitro, GFP-expressing A549 cells were seeded in a Corning 12-well plates at the density of 8 × 10^4^ cells per well, and cultured at 37 °C in 5% CO_2_ with a humidified atmosphere overnight. Thereafter, DL-/L-/D-Au NPs@mPEG@siGFP were added at a 0.2 mg/mL dose. The GFP expression changes of different chiral Au NPs before and after treatment were imaged using a high-content screening. Thereafter, the cells were collected, centrifuged and resuspended in phosphate buffer saline (PBS) buffer solution, the GFP expression in the control and experimental groups was assessed by flow cytometry analysis.

### 2.5 Proteomics analysis

GFP-A549 cells were seeded in 6-well plates at a density of 1 × 10^6^ cells/mL and cultured for 24 h. The cells were then treated with DL-/L-/D-Au RCO@mPEG@siGFP at a final concentration of 2.5 mg/mL, with three replicate wells for each treatment. After an additional 24 h of incubation, the cells were washed twice with ice-cold PBS, harvested entirely by cell scraping, and immediately snap-frozen in liquid nitrogen for transportation. Proteomic analysis was performed by Sangon Biotech Co., Ltd. (Shanghai, China).

### 2.6 Agarose gel electrophoresis

To assess the serum stability of siRNA loaded in the nanocarrier, ‌the free naked siRNA and pre-formed siRNA-loaded nanocarrier complexes (siRNA NPs) were incubated separately in non-inactivated 10% fetal bovine serum (FBS), with the final siRNA concentration kept identical across all groups. The mixtures were maintained in a 37 °C isothermal incubator. At predetermined time points (0, 2, 6, 12, 24 h), 20 μL aliquots were precisely collected from each system. The withdrawn aliquots were immediately mixed with sodium heparin (final concentration, 2%) and incubated for 10 min at room temperature, utilizing the high negative charge of heparin to competitively elute the encapsulated siRNA from the nanocarrier. After thorough mixing with 6× Loading Buffer supplemented with GelRed nucleic acid stain, the samples were loaded onto a 2% agarose gel (TAE buffer) and electrophoresed at a constant 100 V for 20–30 min. Band patterns were imaged and documented using a Bio-Rad GelDoc XR + gel imaging system, and the time-dependent reduction in band intensity is quantified.

### 2.7 Cytotoxicity

To assess whether DL-/L-/D-Au RCO@mPEG-SH@siPD-L1 are toxic to normal cells, the cell counting kit-8 (CCK-8) was used to monitor the cell viability of normal cells and tumor cells. L929, A549, H460 cells were seeded in 96-well plates at the density of 5k cells per well, followed by culture at 37 °C in 5% CO_2_ and a humidified atmosphere overnight. DL-/L-/D-Au RCO@mPEG-SH@siPD-L1 was added at concentrations of 0, 0.2, 0.4, 0.6, and 0.8 mg/mL, respectively, with five replicate wells set for each dose. After treatment for 24 h, 10 μL of CCK-8 reagent was added, and the plate was placed in a 37 °C, 5% CO_2_ incubator for light-protected incubation for 1 h. Finally, the optical density (OD) at 450 nm wavelength was measured with an enzyme-labeled instrument.

### 2.8 Cell proliferation

Cell proliferation was detected using the 5-Ethynyl-2′-deoxyuridine (EdU) kit. A549 and H460 cells were seeded at a density of 8 × 10^4^ cells per well in a Corning 12-well plate and cultured in a humidified environment at 37 °C and 5% CO_2_ overnight. Then, 0.2 mg/mL of DL-/L-/D-Au RCO@mPEG-SH@siPD-L1 was added to treat different cells. After 24 h, the cells were treated according to the instructions of the kit and finally fluorescence detection was performed.

### 2.9 Colony formation assay

The effects of different concentrations of DL-/L-/D-Au RCO@mPEG-SH@siPD-L1 on the proliferation ability of tumor cells were further evaluated through colony formation experiments. The exponentially growing A549 and H460 cells were seeded at a density of 1.6 × 10^5^ cells per well in a 6-well plate and cultured in a humidified environment at 37 °C and 5% CO_2_ overnight. Then, different concentrations of DL-/L-/D-Au RCO@mPEG-SH@siPD-L1 (0, 0.2, 0.4, 0.8 mg/mL) were added successively to treat the different cells. After incubation for 24 h, a new Dulbecco's Modified Eagle Medium (DMEM) was replaced, and the cells were incubated at 37 °C and 5% CO_2_ for 10 days until distinct colonies of individual cells could be observed. Subsequently, the cells' colonies were fixed with 4% paraformaldehyde at room temperature for 15 min, and the fixative was removed. The cells were then stained with 0.1% (*w/v*) crystal violet staining solution for 25 min. The cell colony images were captured using a mobile phone with high-resolution camera, and the cell colony numbers were counted using Image-J software.

### 2.10 Cell uptake

To investigate whether the chirality of nanoparticles influences cellular uptake, a high-content cell analysis system and flow cytometry were employed to assess the uptake of different chiral Au NPs. GFP-A549 cells were seeded in a Corning 12-well plate at the density of 8 × 10^4^ cells per well and cultured at 37 °C in 5% CO_2_ and a humidified atmosphere for 24 h. Then, L/D-Au RCO@mPEG@siCy5 (0.2 mg/well) were dispensed into cells, and the cells were further incubated in the incubator for 3h, 8h, 12h and 24h.

### 2.11 RT-qPCR analysis

H460 and A549 cells were seeded on a 6-well plate at 1.6 × 10^5^ cells per well and cultured in a humidified environment at 37 °C and 5% CO_2_ overnight. After being treated with DL-/L-/D-Au RCO@mPEG-SH@siPD-L1 for 24 h, cells were washed by PBS and the intracellular total RNA was extracted by Trizol. The mRNA was reverse-transcribed by Evo M-MLV reverse transcription pre-mix kit (containing gDNA Eraser). Quantitative polymerase chain reaction (PCR) analysis was performed using the SYBR Green Pro Taq HS pre-mix qPCR kit.

### 2.12 Immunofluorescence staining

The H460 and A549 cells were seeded on a density of 8 × 10^4^ cells per well in a Corning 12-well plate and cultured in a humidified environment at 37 °C with 5% CO_2_ for 24 h. After being treated with DL-/L-/D-Au RCO@mPEG-SH@siPD-L1 for 24 h, a fixative was added to the cells for 15 min, followed by three times washing with PBS buffer for 5 min each time. Then, a permeation agent was added, and three times washing with PBS buffer for 5 min each time were conducted. Subsequently, a blocking solution was added at room temperature for 30 min. Three PBS washes were performed for 5 min each time, and the cells were co-incubated with the PD-L1 primary antibody overnight. Three PBS washes were conducted, and the cells were incubated with the fluorescent secondary antibody at 37 °C for 1 h. Three PBS washes were performed again, and the unbound fluorescent secondary antibody was removed. Then, an anti-fluorescence quenching agent mounting solution containing 4′,6-diamidino-2-phenylindole (DAPI), was added for incubation at room temperature for 5 min before the fluorescence images were captured on the machine.

### 2.13 Animals

Female BALB/c-nu mice were purchased from Chongqing Ensvil Laboratory Animal Sales Company. The care of the animals and the experiments were conducted in accordance with the approval of the Animal Experiment Ethics Committee. After obtaining the approval from the Experimental Animal Center of Southwest University, the animal experiment protocol was adopted.

### 2.14 Tumor model and treatment in vivo

To construct a tumor implantation model. The expanded H460 cells were collected and centrifuged to resuspend in PBS. Then, at a density of 5 × 10^6^ cells per mouse, they were subcutaneously injected into the right legs of 4-5-week old BALB/c-nu mice. The tumor size and the weight of the mice were monitored regularly. When the tumor size reached 50- 00 mm^3^, it was divided into four groups (n = 5), and was administered subcutaneously to mice in three doses: 50 μL of normal saline, DL-Au RCO@mPEG-SH@siPD-L1, L-Au RCO@mPEG-SH@siPD-L1, and D-Au RCO@mPEG-SH@siPD-L1 (0.4 mg of per mouse). Subsequently, the growth of the tumors was recorded by measuring the vertical diameter of the tumors using a caliper, and the calculation formula was: volume = (tumor length) × (tumor width)^2^/2. At the end of the experiment, tumor tissues were collected from all mice and photographed.

### 2.15 Serum sample collection and biochemical index detection

At 24 h post-administration, blood samples were harvested from the orbital venous plexus of mice, and whole blood was aliquoted into 1.5 mL anticoagulant-free centrifuge tubes. The samples were left at room temperature for 1–2 h to achieve complete natural coagulation, then centrifuged at 4 °C and 3000–4000 rpm for 10–15 min. The supernatant serum fraction was carefully aspirated and transferred to a fresh EP tube for downstream analysis. A panel of serum biochemical parameters was quantified using an automatic biochemical analyzer (Hitachi 7600). Sample loading and detection workflows were executed strictly following the instrument's standardized operating procedure. Throughout all measurements, the instrument automatically completed reaction setup, colorimetric detection and result calculation through its integrated optical system. Certified reference serum with a certified concentration value was used for full-system calibration prior to sample testing, ensuring the accuracy and reproducibility of all generated data.

### 2.16 Determination of Au content in tissues by inductively coupled plasma mass spectrometry

Prior to use, the polytetrafluoroethylene liner of the sealed high-pressure digestion vessel was pre-cleaned by soaking in 20% v/v nitric acid solution for 24 h, followed by thorough rinsing with ultrapure water. The liner was then filled with 3.0 mL of concentrated nitric acid and heated at 105 °C for over 1 h, which was rinsed again with ultrapure water and dried for subsequent work.

At 24 h post-administration, major organs were harvested from mice, rinsed, and homogenized. A precisely weighed aliquot of each homogenate (0.1–0.2 g, exact mass recorded) was transferred into a 100 mL pre-treated digestion liner. For complete digestion of gold elements, 10.0 mL of freshly prepared aqua regia (HNO_3_: HCl = 1:3) was added to each liner. The digestion assembly was fully sealed and placed in an oven for programmed heating: 2 h at 100 °C, 2 h at 120 °C, 2 h at 140 °C, and 2 h at 170 °C. After digestion, the vessels were cooled to room temperature. The inner lid of each liner was rinsed with a small volume of ultrapure water, and all rinses were combined with the digested solution inside the liner. The digested samples were then heated on an electric hot plate at 140–160 °C for acid evaporation, until the residual volume was reduced to approximately 1.0 mL. The concentrated digestate was rinsed 3–5 times with 1–2% v/v aqua regia, and all washings were transferred to a 5 mL plastic volumetric flask. The solution was brought to the final volume with ultrapure water, homogenized, and prepared for subsequent inductively coupled plasma mass spectrometry (ICP-MS) analysis.

An external standard calibration strategy was adopted for absolute quantification. A certified Au reference standard was serially diluted using 1–2% aqua regia (matched to sample matrix acidity) to generate a linear calibration series covering 0, 0.01, 0.05, 0.1, 0.5, 1, and 2 μg/L. Heart, liver, spleen, lung and kidney tissue samples from four mouse groups (PBS, DL, DA, LA) were processed in parallel following the identical protocol described above. The instrument software automatically generated the calibration curve and output the measured Au concentration (C_instrument, μg/L). The final Au content in each tissue was calculated using the formula: C_tissue (ng/g) = C_instrument × V_dilution (mL)/m_tissue (g).

### 2.17 Statistical analysis

All data are presented as mean ± SEM from three independent experiments (n = 3). For comparisons between more than two groups, one-way ANOVA followed by Dunnett's multiple comparisons test was used, with each treatment group compared with the control group. P < 0.05 was considered statistically significant. Significance levels are indicated as *P < 0.05, **P < 0.01, and ***P < 0.001. All statistical analyses were performed using GraphPad Prism 9.0 (GraphPad Software, San Diego, CA, USA).

## 3 Results and discussion

### 3.1 Characterization of intrinsically chiral Au NPs with controlled morphology

Au RCO and Au Cube nanoparticles were prepared using a bottom-up seed-mediated approach to investigate the influence of particle morphology on delivery efficiency [40], [41], [42]. The resulting nanoparticles were characterized by scanning electron microscopy (SEM), which revealed a multifaceted structure for Au RCO and a well-defined cubic geometry for Au Cube (Fig. S1).

Using these two morphologically distinct Au NPs as seeds, cysteine (Cys) enantiomers (Fig. S2) was introduced as chiral inducer during particle growth at equivalent concentrations. The resulting nanoparticles exhibited nearly perfect mirror-image circular dichroism (CD) spectra in the 200-1000 nm range, whereas the DL-Au NPs showed no significant CD signal under the same conditions (Fig. 1A). Further spectral analysis revealed that L- and D-Au RCO displayed characteristic CD peaks near 600 nm (±400 mdeg), while the Au Cube variants exhibited peaks around 650 nm with higher intensities approaching ±600 mdeg, confirming the successful preparation of two distinct classes of gold nanomaterials with well-defined chiroptical properties.

**Fig. 1:**
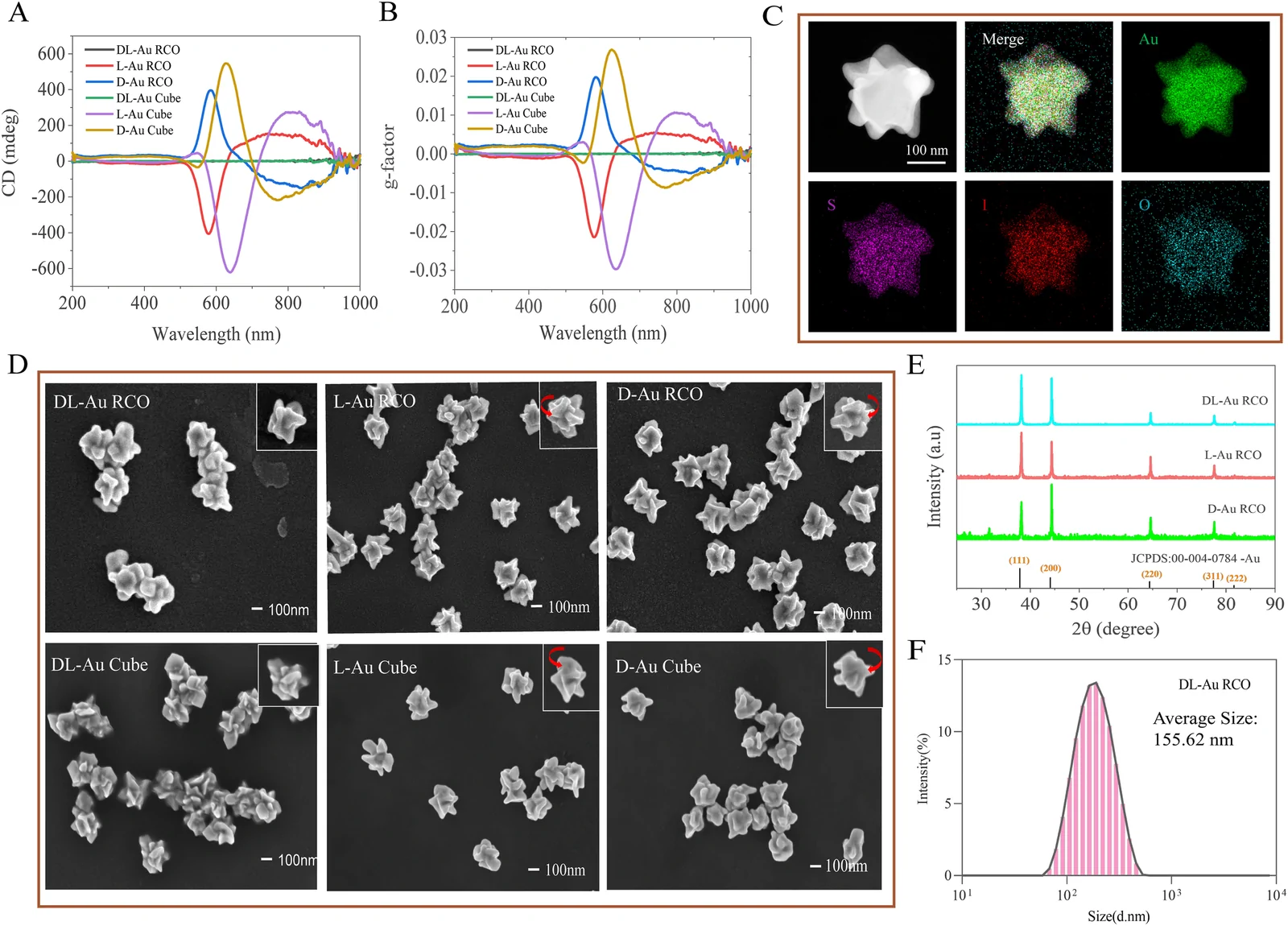
Characterization of intrinsically chiral Au NPs. (A) CD and (B) *g*-factor spectra of DL-/L-/D-Au NPs. (C) Elemental mapping of a single D-Au RCO. (D) SEM images of DL-/L-/D-Au RCO and DL-/L-/D-Au Cube. (E) X-ray diffraction pattern of DL-/L-/D-Au RCO. (F) Dynamic light scattering analysis of DL-Au RCO.

Quantitative evaluation of the chiroptical activity was performed by calculating the dissymmetry factor (*g*-factor) (Fig. 1B). DL-Au NPs exhibited a *g*-factor close to zero with no significant chiral response, confirming that the presence of a single chiral ligand is essential for inducing chiroptical activity. In contrast, L- or D-Au NPs displayed pronounced *g*-factor peaks, which were highly dependent on the geometric configuration of the nanostructures. Specifically, Au RCO exhibited symmetric response peaks of opposite signs at approximately 580 nm, consistent with enantiomeric behavior. Notably, Au Cube exhibited a stronger chiral response, with intense mirror-symmetric peaks at approximately 650 nm and an additional broad feature in the range of 800–900 nm. The stronger chiroptical response of Au Cube compared to Au RCO may arise from the different surface facet compositions and cysteine adsorption geometries on cubic versus rhombicuboctahedral architectures, which influence the local electromagnetic field enhancement and chiral coupling efficiency.

To characterize the elemental composition of chiral Au NPs, elemental mapping was performed using high-resolution transmission electron microscopy (HRTEM). Analysis of DL-/L-/D-Au RCOs revealed the presence of four elements in the order Au > S > I > O (Fig. S3A–B and Fig. 1C), indicating a direct involvement of surface-bound cysteine in directing chiral growth. The presence of iodine (I) likely originates from the gold precursor, consistent with previous reports on seed-mediated growth of Au nanostructures. SEM imaging was employed to examine the morphology of the chiral nanoparticles (Fig. 1D). Red arrows in the images indicate the chiral orientation of individual particles: DL-Au NPs appeared achiral, whereas L-Au NPs and D-Au NPs exhibited left-handed and right-handed characteristics, respectively, forming a mirror-image relationship consistent with the CD data. These observations are consistent with all diffraction peaks matching standard reference data (JCPDS No. 004-0784, Fig. 1E). Dynamic light scattering (DLS) measurements revealed that the hydrodynamic diameters of DL-Au RCO, L-Au RCO, and D-Au RCO were 155.62 nm, 153.48 nm, and 153.19 nm, respectively (Fig. 1F and S4A–B), which were smaller than those of the corresponding Au Cube variants (DL-Au Cube: 172.34 nm, L-Au Cube: 169.02 nm, D-Au Cube: 175.40 nm; Fig. S4C–E). The slightly smaller hydrodynamic diameter of Au RCO compared to Au Cube is consistent with their geometric differences, as the multifaceted RCO structure presents a more compacted projection than the cubic architecture. Critically, both morphologies fall within the 150–180 nm range, which is a size regime known to support efficient cellular uptake via endocytosis, minimizing the potential confounding effect of size on the observed morphology-dependent delivery efficiency.

### 3.2 Chiral characterization and siRNA loading performance after minimal surface modification

The above intrinsically chiral Au NPs were surface functionalized with a classical salt aging protocol [43], [44]. To validate the feasibility of this chiral nanoplatform for gene delivery, DL-/L-/D-Au NPs were first conjugated with siGFP as a model gene, and systematically evaluated the siGFP loading characteristics of Au NPs with different chiralities and morphologies. Surface modification and siGFP loading were accomplished via a two-step strategy. First, Au NPs were functionalized with methoxypolyethylene glycol thiol (mPEG-SH) [45] to enhance aqueous solubility and colloidal stability while reducing biotoxicity. Second, a thiol group (-SH) was introduced at the 3′-terminus of the siGFP sequence. Based on the specific Au-S covalent bond interaction [46], [47], efficient immobilization of siGFP onto the Au NP surface was achieved, ultimately yielding the DL-/L-/D-Au NPs@mPEG-SH@siGFP composite delivery system.

Fourier-transform infrared (FTIR) spectroscopy was first employed to characterize Au NPs after mPEG-SH modification. It was found that the FTIR spectrum of DL-Au RCO@mPEG-SH exhibited distinct differences from that of unmodified DL-Au RCO, with newly emerged characteristic absorption bands corresponding to mPEG-SH (Fig. 2A and S5A–B), confirming the successful surface functionalization of Au RCO with mPEG-SH. Subsequently, zeta-potential measurements were performed to assess siGFP loading on the nanoparticle surface and optimize its loading concentration. the initial positive surface charge of DL-/L-/D-Au RCO was fully neutralized at 30 nM siGFP, resulting in a moderately negative zeta potential of the composite. This 30 nM concentration was thus identified as the optimal siGFP binding concentration for DL-/L-/D-Au RCO (Fig. 2B). The unmodified DL-Au RCO, L-Au RCO and D-Au RCO showed zeta potentials of 35.73, 35.87 and 36.53 mV, respectively. Their surface potentials shifted after mPEG-SH modification, and further decreased to −2.63, −3.44 and −7.63 mV after siGFP loading. DL-/L-/D-Au Cube were also dual-modified under identical conditions for zeta-potential characterization. The Au Cube series showed a similar potential trend to Au RCO, but the Au RCO groups maintained relatively more negative potentials, demonstrating more efficient siRNA loading on Au RCO (Fig. S6A–B).

**Fig. 2:**
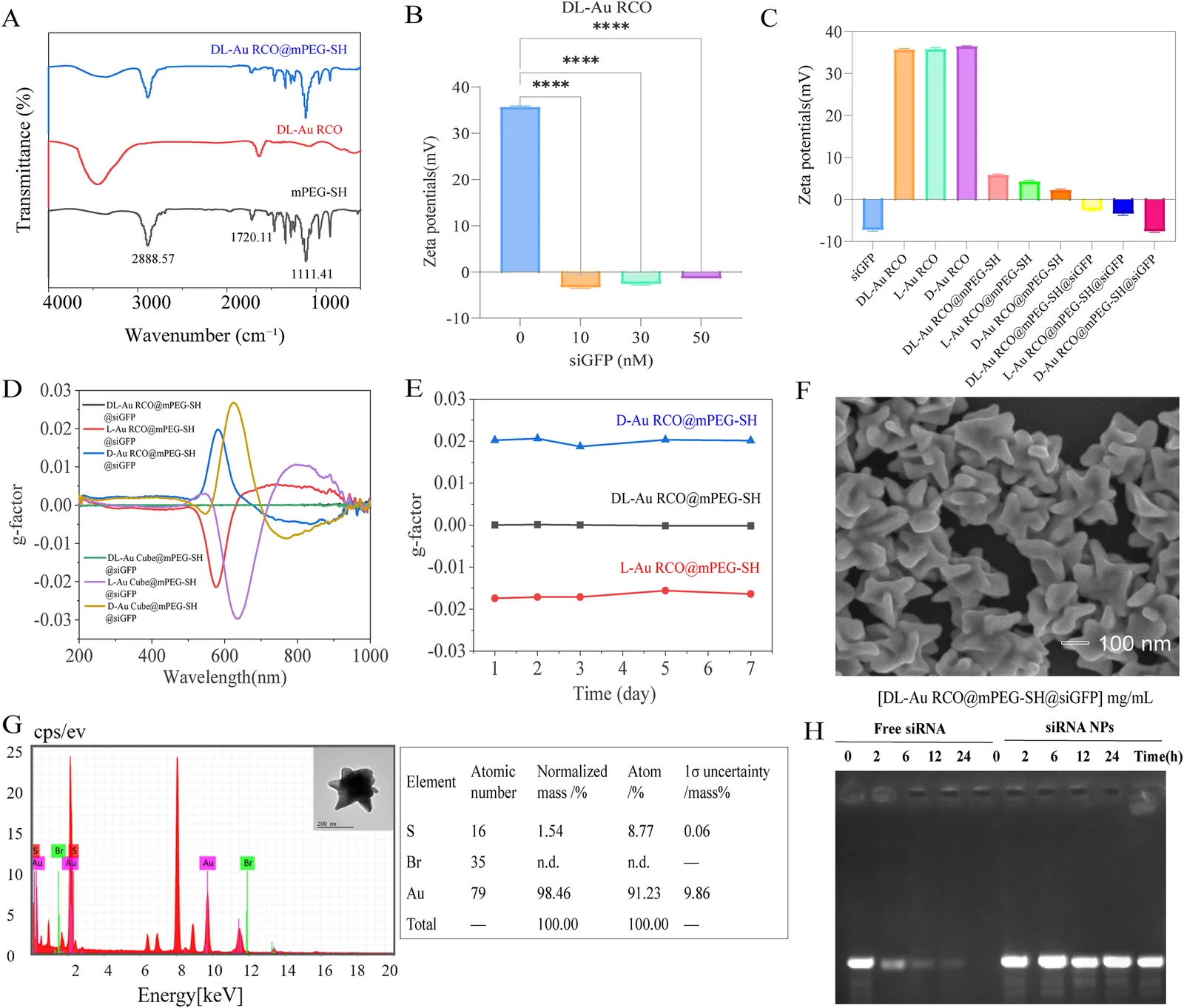
Chiral characterization and siRNA loading. (A) FTIR spectra of mPEG-SH, DL-Au RCO and DL-Au RCO@mPEG-SH. (B) Zeta potential of the DL-Au RCO@mPEG-SH after modification with 0, 10, 30 and 50 nM siGFP, respectively. (C) Zeta potential change of DL-/L-/D-Au RCO after modification. (D) *g*-factor spectra of DL-/L-/D-Au RCO/Cube@mPEG-SH@siGFP. (E) *g*-factor changes of DL-/L-/D-Au RCO@mPEG-SH at different time points. (F) SEM images of modified D-Au RCO. (G) EDS analysis of DL-Au RCO@mPEG-SH. n.d., not detected (Br signal below the detection limit under the acquisition conditions). (H) Gel electrophoresis stability analysis of D-Au RCO@mPEG-SH@siGFP under serum conditions.

Moreover, to verify whether the chirality-dependent delivery performance arises from distinct siRNA binding behaviors, microscale thermophoresis (MST) was employed to quantify the binding affinity between L-/D-Au RCO and siGFP, and the corresponding dissociation constant (Kd) was calculated [48], with 0.099 μM for D-Au RCO and 0.142 μM for L-Au RCO (Fig. S8). These results indicate that D-Au RCO binds siGFP more strongly than L-Au RCO, representing an approximately 1.4-fold binding affinity, which may contribute to its more efficient siRNA loading and targeted delivery. It is worth noting that the absolute difference in binding affinity between the two chiral Au NPs remains moderate; as elaborated in the following section, further increasing the *g*-factor is expected to effectively amplify this chirality-dependent effect.

CD spectroscopy was employed to assess the impact of the modification process on the chiral properties of the nanoparticles and to characterize the DL-/L-/D-Au NPs@mPEG-SH@siGFP composites (Fig. S9). The modified composites retained prominent chiral signals with an intensity of ∼400 mdeg, which was essentially consistent with the chiral signal intensity prior to modification. The *g*-factor was subsequently adopted as a unified quantitative metric to systematically compare the chiroptical responses of gold nanoparticles with distinct handedness and morphologies. After surface functionalization with mPEG-SH and subsequent loading of siGFP, DL-/L-/D-Au NPs@mPEG-SH@siGFP retained their well-defined, characteristic chiroptical signatures. This observation confirms that neither the mPEG-SH surface modification nor the siGFP loading process disrupts the intrinsic structural chirality of the parent nanoparticles (Fig. 2D). To further rigorously validate the robustness of this preserved chiral property, the long-term chiroptical stability of DL-/L-/D-Au RCO@mPEG-SH was continuously monitored over a 7-day observation period (Fig. S10). The *g*-factor was utilized as a normalized analytical parameter to quantify the evolution of the gold-associated chiral signal, with time-point measurements systematically performed on day 1, day 2, day 3, day 5, and day 7 post-preparation. As shown in Fig. 2E, all three groups exhibited generally stable chiroptical signals throughout the observation period, with no obvious attenuation, demonstrating good chiroptical stability after mPEG-SH modification. This outcome demonstrates that the adopted surface functionalization strategy preserves the intrinsically chiral characteristics of the Au NPs while successfully endowing the nanomaterials with enhanced hydrophilicity and colloidal stability, providing a critical safeguard for their application in biological contexts.

Taking DL-/L-/D-Au RCO modification as a representative example, SEM characterization revealed that the modified particles exhibited a densely packed configuration without noticeable aggregation (Fig. 2F, Fig. S11A–B). Since CTAB was involved in the synthesis and temporary storage of these chiral gold nanomaterials, and to rule out residual CTAB as a potential source of surface-associated toxicity, transmission electron microscopy-coupled energy-dispersive X-ray spectroscopy (TEM-EDS) point analysis was subsequently performed to characterize the elemental composition of mPEG-SH-modified chiral Au RCO (Fig. 2G, Fig. S12A–B). Under the EDS acquisition conditions established for this study, the characteristic bromine signal remained below the instrumental detection limit, and no distinct Br-specific peak was resolved in the collected spectra. These observations indicated that no detectable CTAB residues were present on the surface of the functionalized nanomaterials. Because uncomplexed free siRNA is inherently susceptible to nuclease-mediated degradation in serum, agarose gel electrophoresis was further implemented to evaluate the serum stability of siGFP after loading onto the gold nanoparticle scaffold. As shown in Fig. 2H, nanoparticle-conjugated siGFP exhibited no appreciable degradation following serum incubation. This result demonstrated that loading onto the gold nanoparticles improved the stability of siRNA in biological environments and provided effective protection for subsequent intracellular delivery.

### 3.3 Evaluation of siRNA delivery efficacy and proteomic analysis of functionalized intrinsically chiral Au NPs

Capitalizing on the successful conjugation of siGFP onto DL-/L-/D-Au NPs, the biosafety of the nanocomplex using normal fibroblast L929 cells was evaluated as a model. Cell viability was assessed via CCK-8 assay (Fig. 3A and S13A-B), which indicated that DL-/L-/D-Au RCO@mPEG-SH@siGFP elicited no significant cytotoxicity toward L929 cells at a concentration of 0.4 mg/mL. Even at the higher dose of 0.8 mg/mL, only a slight decrease in viability was observed in the D-Au RCO@mPEG-SH@siGFP treatment group, with cell survival rates remaining around 90%. These findings highlight the favorable biocompatibility and low cytotoxicity of the nanoformulation.

**Fig. 3:**
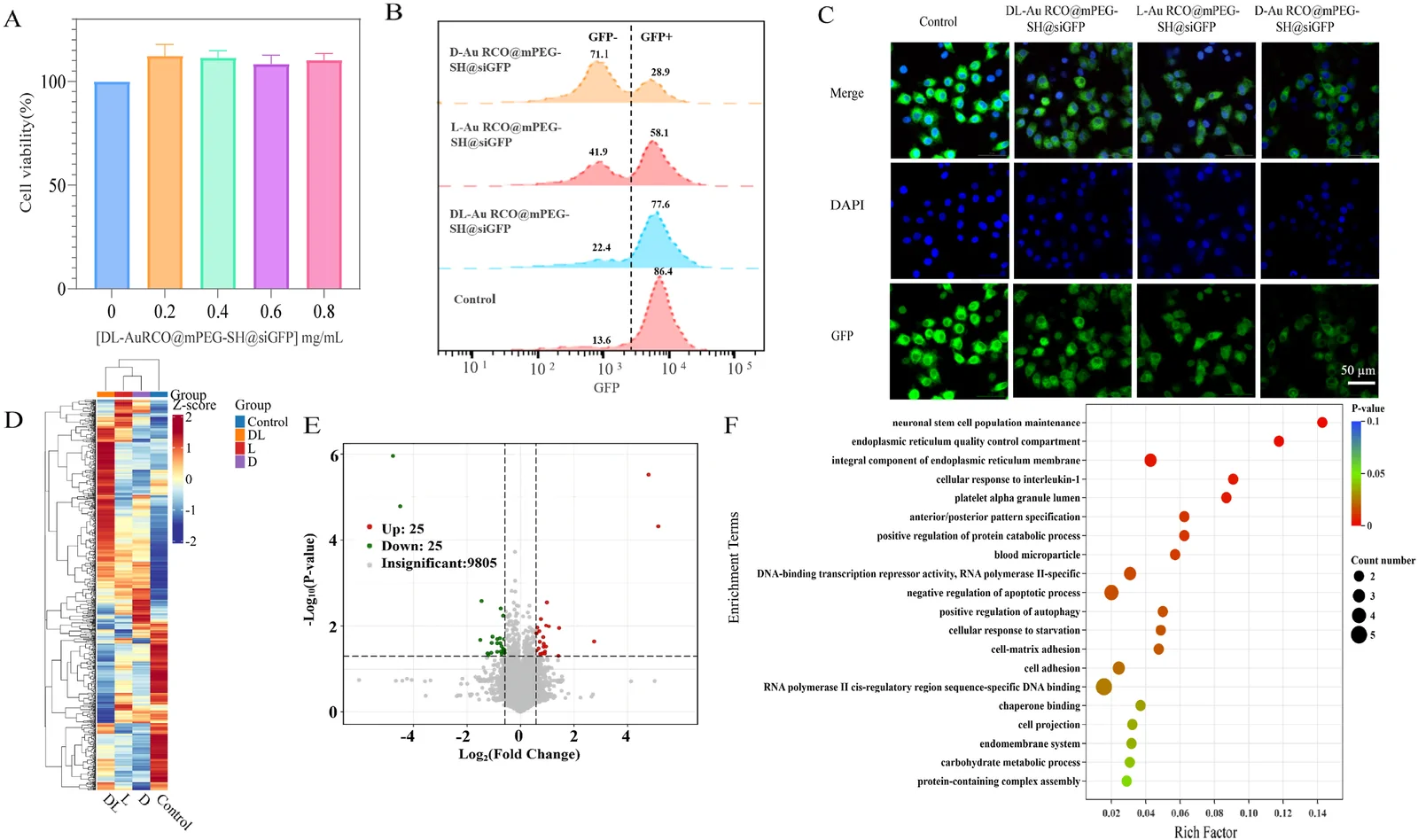
siRNA delivery and proteomic analysis of functionalized intrinsically chiral Au NPs. (A) Cell viability of L929 cells after treatment with DL-Au RCO@mPEG@siGFP. Error bars represent the standard error of the mean (SEM, n = 3). (B) Flow cytometric analysis of GFP fluorescence intensity in GFP-A549 cells after 24 h of co-incubation with DL-/L-/D-Au RCO@mPEG-SH@siGFP. (C) Fluorescence images of GFP-A549 cells after treatment with DL-/L-/D-Au RCO@mPEG-SH@siGFP for 24 h. Scale bar: 100 μm. (D) Hierarchical clustering heatmap of differentially expressed proteins among the Control, DL-, L-, and D-Au RCO groups. Relative protein abundance values from biological replicates in each group were Z-score normalized and clustered according to their expression patterns. (E) Quantification of differentially expressed proteins between the D- and L-Au RCO groups. Using the criteria of FC ≥ 1.5 or FC ≤ 0.667 and P ≤ 0.05, a total of 50 differentially expressed proteins were identified, including 25 upregulated and 25 downregulated proteins. (F) GO functional enrichment analysis of differentially expressed proteins between the D- and L-Au RCO groups.

Subsequently, its gene silencing efficacy was further investigated. The intracellular delivery efficiency and GFP silencing capacity of DL-/L-/D-Au NPs@mPEG-SH@siGFP were evaluated using GFP-A549 cells, a stable GFP-expressing A549 cell line. Following incubation with chiral Au NPs@mPEG-SH@siGFP featuring different handedness and crystal morphologies, GFP expression levels were characterized both quantitatively by flow cytometry and qualitatively by fluorescence imaging. Flow cytometry was first employed to quantitatively assess the gene silencing efficiency of siRNA delivered by these chiral nanomaterials across different handedness (Fig. 3B and Fig. S14). After 24 h of co-incubation, all Au NPs@mPEG-SH@siGFP composites reduced GFP expression to varying degrees relative to the untreated and negative control groups. Notably, D-Au NPs@mPEG-SH@siGFP induced significantly greater GFP downregulation than DL- and L-Au NPs@mPEG-SH@siGFP, underscoring the critical role of chiral handedness in modulating silencing efficacy. Beyond chirality, remarkable differences in silencing efficiency were also observed among composites with identical handedness but distinct crystal morphologies. Specifically, D-Au RCO@mPEG-SH@siGFP achieved a silencing efficiency of 71.1%, which was 1.48-fold of D-Au Cube@mPEG-SH@siGFP (48.2%), further indicating that crystal structure serves as an additional determinant of gene silencing performance.

Complementing the quantitative flow cytometry results, fluorescence imaging analysis qualitatively confirmed these observations. As shown in Fig. 3C, D-Au RCO@mPEG@siGFP treatment led to a substantial reduction in GFP fluorescence compared with the control, DL- and L-Au RCO@mPEG@siGFP. A similar decreasing trend was observed in Au Cube@mPEG@siGFP-treated cells (Fig. S15); however, the overall fluorescence intensity remained higher than RCO-treated groups, further confirming the superior silencing efficacy of the RCO architecture.

To further elucidate the interrelationships among chirality, particle morphology, and physical size, a series of chiral gold nanoparticles with systematically varied morphologies and distinct chiral properties were fabricated. It was founf that D(1,2,3)-Au RCO and D-Au Cube displayed well-resolved, distinct chiral characteristic peaks and measurable dissymmetry factors (Fig. S16A). Building on this foundation, the identical dual-modification strategy was implemented to achieve successful siGFP loading across all nanoparticle variants, after which their respective gene silencing efficiencies were quantitatively evaluated. Compared to the untreated control group, the D(1,3)-Au RCO@mPEG-SH@siGFP formulation, which possessed a nearly equivalent *g*-factor but a 20 nm redshifted chiral characteristic peak, produced a comparable level of GFP gene silencing (Fig. S16B). In contrast, the D2-Au RCO@mPEG-SH@siGFP group, whose chiral characteristic peak was centered at 580 nm but carried a substantially lower *g*-factor, retained prominent green fluorescence signal. Notably, despite the relatively greater *g*-factor measured for bare D-Au Cube, the D-Au Cube@mPEG-SH@siGFP construct did not yield an improved GFP silencing effect. Thus, these results suggest that the specific pairing of intrinsic D-type chirality with the RCO particle morphology yields the optimal gene silencing performance. This finding lays a critical foundation for establishing a direct, empirically supported correlation between the co-optimization of chirality and morphology, and the resulting enhancement in functional nucleic acid delivery efficacy.

Following the observed differences in cellular uptake and siRNA delivery across chiral Au RCO variants, label-free quantitative proteomics was performed to profile chirality-dependent cellular responses. All cells were treated with siRNA-free DL-, L-, or D-Au RCO to eliminate confounding effects from siRNA-mediated gene silencing. Global protein abundance was compared across Control, DL, L and D groups. Significant differentially expressed proteins (one-way ANOVA, P ≤ 0.05) were normalized by Z-score and clustered, producing clearly segregated expression patterns in the heatmap (Fig. 3D). These proteins were primarily enriched in translation, ribosomal biogenesis, mitochondrial, endoplasmic reticulum and chromatin organization pathways (Fig. S17A). Pairwise comparison against Control revealed distinct functional signatures for each chiral group: D-Au RCO altered translation, ribosome and chromatin regulatory pathways; L-Au RCO perturbed membrane localization, molecular transport, protein modification and cell cycle; DL-Au RCO modulated intracellular vesicular trafficking, glycosylation and phospholipid transport (Fig. S17B–D). Direct comparison between D- and L-Au RCO identified 50 significantly differentially expressed proteins, with fully segregated expression profiles confirmed by hierarchical clustering (Fig. 3E, Fig. S18). These proteins were enriched in endoplasmic reticulum compartments, membrane-bound organelles, cell adhesion, proteostasis and transcriptional regulation (Fig. 3F). These data confirm that distinct chiral Au RCO variants produce divergent siRNA delivery efficiencies, accompanied by chirality-specific proteome remodeling. This molecular-level evidence directly corroborates the previously documented differences in cellular internalization and functional delivery across the nanoparticle series.

### 3.4 In vitro antitumor efficacy of siPD-L1 delivered by chiral Au NPs

Building on the mechanistic insights into chirality-dependent cellular internalization and intracellular regulatory effects obtained in preceding experiments, subsequent efforts were directed toward translating these fundamental findings into therapeutic applications. Sustained aberrant overexpression of PD-L1 constitutes a major persistent barrier to effective tumor immune modulation, and this therapeutic bottleneck is further compounded by substantial interpatient heterogeneity [49]. Given the validated high efficiency of chiral gold nanoparticles as siRNA delivery platforms, the DL-/L-/D-Au RCO@mPEG-SH@siPD-L1 composite nanoformulation was fabricated via co-incubation of DL-/L-/D-Au RCO@mPEG-SH with siPD-L1. This engineered nanoformulation was subsequently co-cultured with a panel of PD-L1-expressing tumor cell lines to systematically evaluate its in vitro antitumor performance.

Specifically, DL-/L-/D-Au RCO@mPEG-SH@siPD-L1 was co-incubated with the PD-L1-positive tumor cell lines A549 (lung adenocarcinoma) and H460 (non-small cell lung cancer) for 24 h. The CCK-8 assay was then employed to assess the inhibitory effect on cell proliferation. Among the three chiral formulations, D-Au RCO@mPEG-SH@siPD-L1 consistently exhibited the strongest inhibitory effect across all cell lines tested (Fig. 4A–C). In A549 cells, D-type treatment reduced cell viability to approximately 20% at a dose of 0.2 mg/mL, whereas DL- and L-type formulations showed only moderate inhibition, maintaining cell survival rates of nearly 60% (Fig. S19A–C). Dose-response experiments further confirmed that the antiproliferative effect of D-Au RCO@mPEG-SH@siPD-L1 was dose-dependent in both cell lines across the 0.05–0.8 mg/mL range. Notably, H460 cells, which express relatively higher levels of PD-L1 compared to A549, exhibited enhanced sensitivity to D-type treatment, with cell viability reduced to approximately 40% at the same dose. This observation suggests that the therapeutic efficacy of the D-RCO formulation may correlate with baseline PD-L1 expression levels, highlighting the potential of this platform for biomarker-guided therapeutic application.

**Fig. 4:**
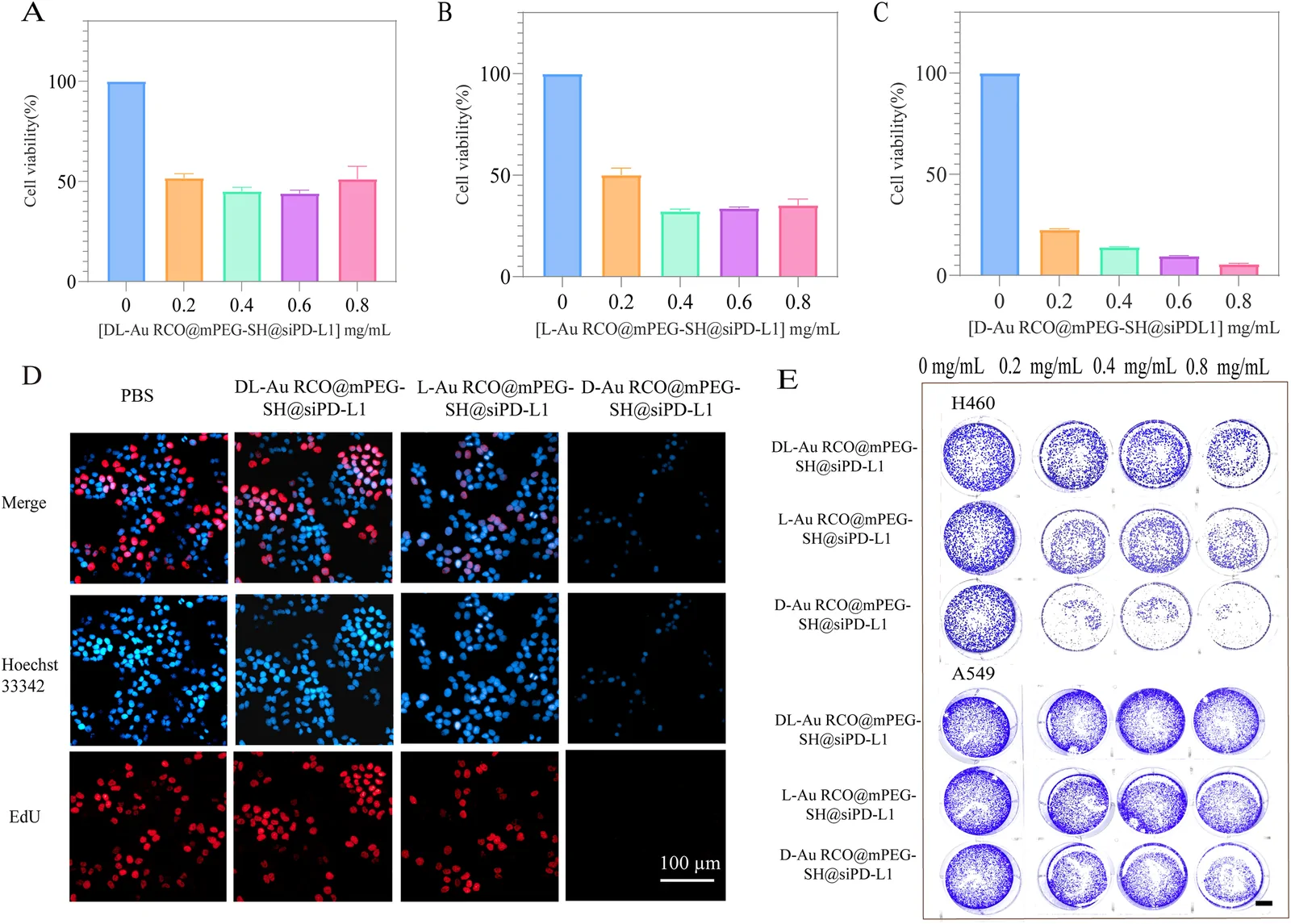
Antitumor effect of DL-/L-/D-Au RCO@mPEG-SH@siPD-L1 in vitro. (A-C) Cell viability of H460 cells after incubation with DL-/L-/D-Au RCO@mPEG-SH@siPD-L1. Error bars represent SEM (n = 3). (D) Fluorescence imaging of EdU-positive H460 cells following incubation with DL-/L-/D-Au RCO@mPEG-SH@siPD-L1. Scale bar: 100 μm. (E) Photographic differences in colony formation of H460 and A549 cells after crystal violet staining. Scale bar: 1 cm.

Further validation of the above hypothesis was carried out using the EdU labeling assay to assess the proliferative status of distinct tumor cell lines. Following co-culture of DL-/L-/D-Au RCO@mPEG-SH@siPD-L1 with H460 cells, EdU labeling revealed pronounced disparities in proliferative activity across groups. D-Au RCO@mPEG-SH@siPD-L1 treatment drastically inhibited cell proliferation, with EdU-positive red fluorescence nearly absent. In contrast, L- and DL-treated groups retained detectable red fluorescence, indicating only moderate suppression (Fig. 4D). Similarly, in A549 cells, D-type treatment exhibited the strongest inhibitory effect among the three chiral formulations, although the overall proliferation inhibition was less pronounced than in H460 cells (Fig. S20). To exclude potential nonspecific effects, a blank vector group, a scrambled siRNA group, and a free siPD-L1 group were included as controls (Fig. S21). The blank vector and scrambled siRNA groups showed proliferation levels comparable to those of the Control group, while the free siPD-L1 group also exhibited no significant inhibition of proliferation, probably owing to its susceptibility to nuclease degradation and inefficient cellular uptake. These results indicate that the pronounced antiproliferative effect of D-Au RCO@mPEG-SH@siPD-L1 originates from the efficient delivery of functional siPD-L1, rather than from the carrier itself or nonspecific siRNA effects.

Additional evidence for the impact of DL-/L-/D-Au RCO@mPEG-SH@siPD-L1 on tumor cell proliferation was provided by a colony formation assay. As illustrated in Fig. 4E, the proliferative capacity of H460 cells was significantly attenuated following treatment with D-Au RCO@mPEG-SH@siPD-L1, with the antiproliferative efficacy following the order: D > L > DL > control group. Although the antiproliferative effect on A549 cells was relatively modest, the inhibitory trend was consistent with that observed in H460 cells. Overall, these complementary experimental data further support the chirality-dependent antiproliferative efficacy of the DL-/L-/D-Au RCO@mPEG-SH@siPD-L1 nanoformulation, with D-handed nanoparticles exhibiting superior performance, particularly in H460 cells, which express relatively higher PD-L1 levels.

### 3.5 Antitumor mechanism and toxicity evaluation of PD-L1 siRNA delivered by L/D-chiral Au NPs

To evaluate the gene-silencing and antitumor effects of siPD-L1 delivered by chiral Au RCO, the mRNA expression levels of *PD-L1* and *STAT3* in H460 and A549 cells were quantified via RT-qPCR. D-Au RCO@mPEG-SH@siPD-L1 significantly downregulated *PD-L1* and *STAT3* mRNA expression in H460 cells compared to the DL- and L-Au RCO@mPEG-SH@siPD-L1 groups, achieving approximately 60% downregulation of *PD-L1* and 55% downregulation of *STAT3*, respectively (Fig. 5A–B). In contrast, the changes in gene expression levels in A549 cells were relatively modest (Fig. S22A-B). Given that *STAT3* silencing and siPD-L1-mediated *PD-L1* suppression have been reported to induce cell apoptosis [50], the mRNA expression of the apoptotic effector cysteine-aspartic acid protease-3 (caspase-3) was further analyzed. Notably, no remarkable activation of *caspase-3* was detected following *STAT3* downregulation (Fig. 5C–S22C). Subsequent Annexin V-FITC/PI double staining flow cytometry assays also revealed an absence of significant apoptotic signals (Fig. S23). These observations suggest that the antiproliferative effect of siPD-L1-mediated gene silencing may occur through non-apoptotic mechanisms, such as cell cycle arrest or senescence, rather than through the activation of classical apoptotic pathways.

**Fig. 5:**
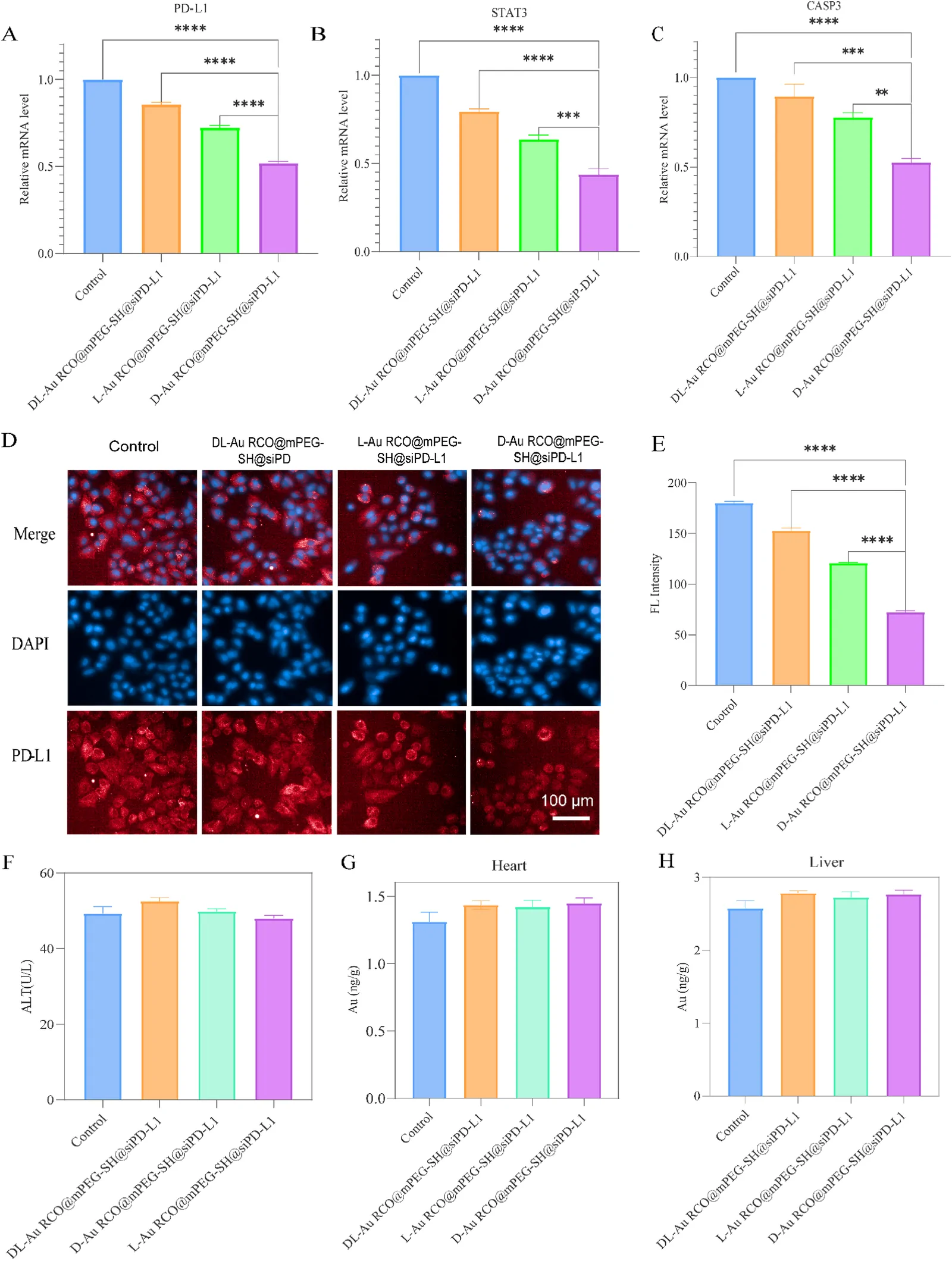
Antitumor mechanism and toxicity evaluation of PD-L1 siRNA delivered by intrinsically chiral Au NPs with different handedness. (A) *PD-L1* (B) *STAT3* and (C)Relative *Caspase-3* mRNA levels in H460 cells after treatment with DL-/L-/D-Au RCO@mPEG-SH@siPD-L1 for 24 h. (D) Immunofluorescence images of H460 cells after treatment with DL-/L-/D-Au RCO@mPEG-SH@siPD-L1 for 24 h. Scale bar: 100 μm. (E) Quantification of average PD-L1 fluorescence intensity of H460 cells from immunofluorescence images. (F) Serum ALT levels in mice after different treatments. (G-H) Au contents in major organs of mice from different treatment groups as quantified by ICP-MS: (G) heart and (H) liver. Error bars represent SEM (n = 3 for A–C and E; n = 5 for F–H). Comparisons among multiple groups were performed using one-way ANOVA followed by Dunnett's multiple comparisons test. *p < 0.05, **p < 0.01 and ***p < 0.001 indicate statistically significant differences.

Subsequently, immunofluorescence (IF) staining was performed to independently assess PD-L1 protein expression in H460 and A549 cells treated with DL-, L-, or D-Au RCO@mPEG-SH@siPD-L1. In H460 cells receiving D-Au RCO@mPEG-SH@siPD-L1 treatment, only a faint red fluorescent signal corresponding to PD-L1 was detectable in the IF micrographs (Fig. 5D). Quantitative analysis of mean fluorescence intensity revealed an approximately 60% reduction in PD-L1 protein abundance in D-treated H460 cells relative to the untreated control group (Fig. 5E), providing orthogonal protein-level validation for the earlier RT-qPCR gene silencing results. These data collectively confirm that Au RCO-mediated siPD-L1 delivery exhibits a pronounced chirality-dependent functional profile, where D-handed nanoparticles effectively downregulate STAT3 signaling via targeted PD-L1 silencing, resulting in robust suppression of tumor cell proliferation. Notably, A549 cells subjected to the identical treatment regimen displayed a comparable magnitude of PD-L1 downregulation following D-type nanoparticle administration, despite having a markedly lower baseline endogenous PD-L1 expression level than H460 cells (Figs. S24–S25). This observation further validates that the siPD-L1-mediated gene silencing effect produces a cell-type-dependent inhibitory response that scales proportionally with the endogenous PD-L1 expression level of the target tumor cells.

After validating in vitro siPD-L1 delivery and antitumor activity, the in vivo biosafety of Au RCO@mPEG-SH@siPD-L1 and gold biodistribution in major organs were assessed. Serum biochemical analysis showed that alanine aminotransferase (ALT), aspartate aminotransferase (AST), blood urea nitrogen (BUN) and creatinine (CREA) levels in all nanoparticle-treated groups remained within normal physiological ranges, with no significant differences from the control group (Fig. 5F, Fig. S26A–C). These results confirm no detectable hepatic or renal toxicity at the tested dose. ICP-MS quantification of gold in the heart, liver, spleen, lung and kidney revealed consistently low tissue gold levels across all groups, which presented no significant accumulation in treated mice relative to controls (Fig. 5G–H and S27A–C). Collectively, serum biochemistry and biodistribution data demonstrate that this chiral nanodelivery system induces no detectable systemic in vivo toxicity under the present working conditions.

### 3.6 In vivo antitumor efficacy and biosafety evaluation

The in vivo therapeutic efficacy of the intrinsically chiral Au RCO-based nanoplatform was tested in a subcutaneous xenograft model of H460 cells in nude mice. Drug administration was performed via intratumoral injection. Mice were randomly divided into four groups (5 mice per group) and treated with: PBS, DL-, D-, or L-Au RCO@mPEG-SH@siPD-L1. The administered dose was 0.4 mg per mouse (approximately 20 mg/kg based on an average mouse body weight of 20 g), with injections given every two days for three cycles over a 12-day experimental period.

Tumor growth monitoring revealed that the D-Au RCO@mPEG-SH@siPD-L1 treatment group exerted a significant inhibitory effect on tumor progression compared to the DL-, L-Au RCO@mPEG-SH@siPD-L1, and PBS control groups, as evidenced by marked reductions in both tumor volume and tumor weight (Fig. 6A–D). Notably, throughout the treatment period, no obvious abnormal fluctuations in body weight were observed in any group (Fig. 6E), indicating that the nanomaterial exhibits no overt acute toxicity at the administered dose. Continuous tracking of tumor volume dynamics further demonstrated that the tumor inhibitory effect of the D-Au RCO@mPEG-SH@siPD-L1 group was consistently superior to that of the other groups (Fig. 6F), confirming its potent and stable in vivo antitumor activity. Collectively, these findings demonstrate the chirality-dependent in vivo antitumor efficacy of D-Au RCO@mPEG-SH@siPD-L1 in a subcutaneous H460 xenograft model, providing a proof-of-concept for its further development as a nanotherapeutic for lung cancer.

**Fig. 6:**
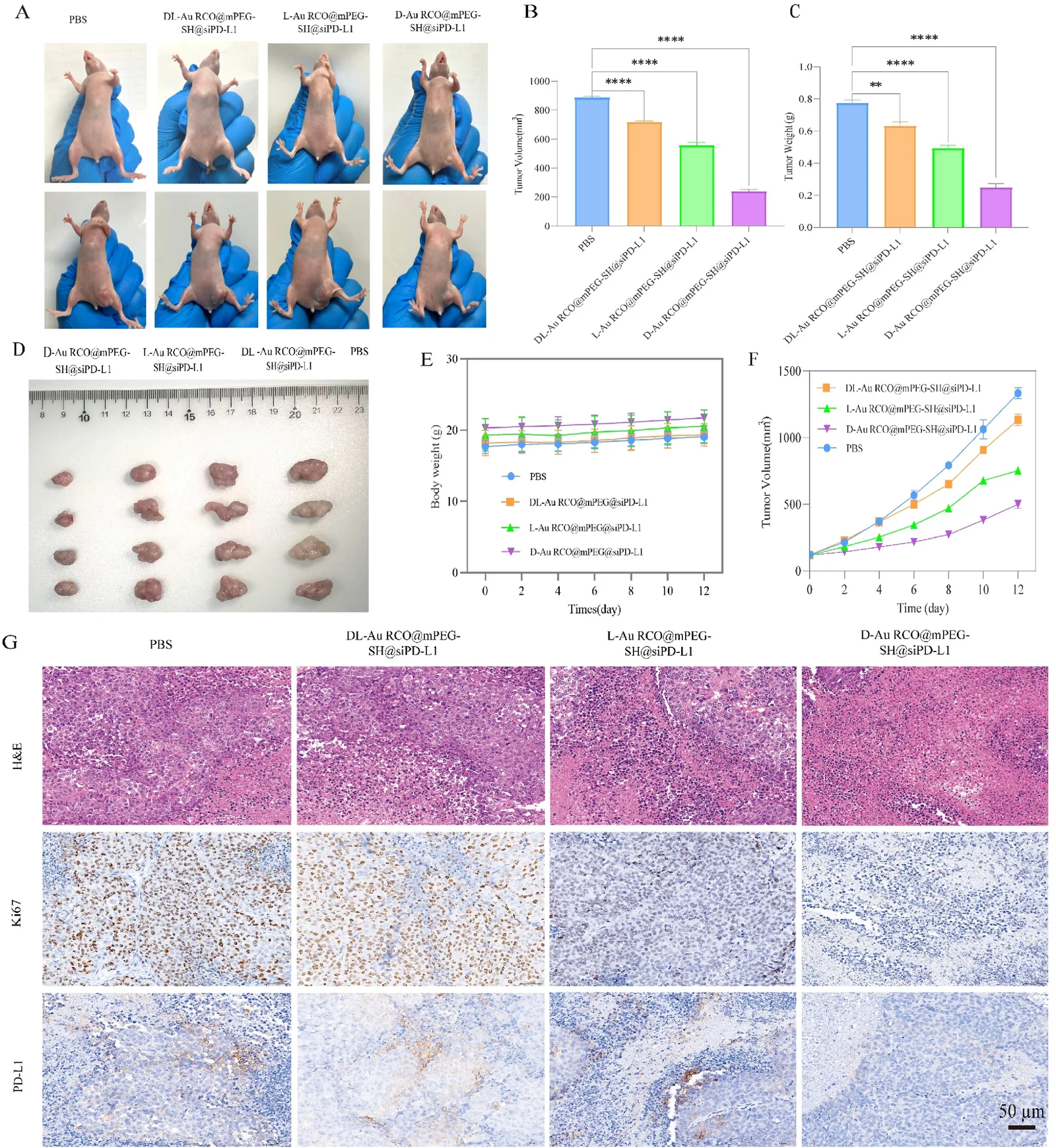
In vivo antitumor effect of DL-/L-/D-Au RCO@mPEG-SH@siPD-L1. (A) Representative photographs of tumor-bearing mice after 12 days of treatment. (B) Tumor volume and (C) weight of H460 tumor-bearing mice treated with different formulations (n = 5 mice). (D) Photographs of the tumor harvested from mice after the final treatment. (E) Body weight and (F) tumor volume changes of treated mice during the therapy for 12 days. (G) H&E staining of tumor sections in all groups after various treatments. Scale bar: 50 μm. Error bars represent SEM (n = 5 mice per group). Comparisons among multiple groups were performed using one-way ANOVA followed by Dunnett's multiple comparisons test. *p < 0.05, **p < 0.01 and ***p < 0.001 indicate statistically significant differences.

The in vivo biosafety of intrinsically chiral Au RCO@mPEG-SH@siPD-L1 was evaluated by hematoxylin and eosin (H&E) staining of major organ sections (heart, liver, spleen, lung, and kidney) from mice across all experimental groups (Fig. S28). No significant pathological abnormalities were observed in any of the examined organs, confirming the favorable biosafety profile of DL-/L-/D-Au RCO@mPEG-SH@siPD-L1. In contrast, H&E staining of tumor tissues revealed marked pathological changes exclusively in the D-Au RCO@mPEG-SH@siPD-L1 treatment group, characterized by pronounced cellular necrosis and structural disorganization (Fig. 6G). These findings further corroborate the chirality-dependent antitumor activity of chiral Au NPs in vivo. Immunohistochemical staining showed that D-Au RCO@mPEG-SH@siPD-L1 significantly downregulated PD-L1 expression in tumor tissues and significantly suppressed tumor cell proliferation, as evidenced by a marked reduction in the Ki67-positive staining rate from ∼60% in the control group to ∼20% in the D-treated group. These results confirm the on-target activity of the siPD-L1 payload and the potent antiproliferative effect of the D-RCO formulation in vivo.

In brief, these findings establish D-Au RCO as a promising gene delivery vector, distinguished by both potent in vivo antitumor efficacy and excellent biosafety. This work provides critical experimental evidence for the local antitumor efficacy of the D-Au RCO nanoplatform, and highlights the need for further evaluation of its systemic delivery efficacy via intravenous administration toward future clinical translation. Moreover, this work further highlights the substantial potential of intrinsically chiral Au NPs for application in precision cancer therapeutics.

## 4 Conclusions

In summary, this work presented the rational fabrication of intrinsically chiral gold nanoparticles with two distinct morphologies (RCO and nanocube), and their systematic evaluation as non-viral siRNA delivery vectors across in vitro cellular models and in vivo tumor-bearing mouse systems. Using a full 2 × 2 factorial experimental design that covered two chiral configurations and two morphologies, the independent and synergistic contributions of these two core engineering parameters were successfully decoupled to gene delivery performance. Functional characterization revealed that D-handed Au NPs exhibit markedly higher cellular internalization efficiency and subsequent gene silencing activity than their L- and DL-Au counterparts, enabling potent, sequence-specific knockdown of target genes. Notably, for nanoparticles with identical handedness, siRNA silencing efficacy was strongly morphology-dependent: the RCO architecture significantly outperformed the cubic counterpart, establishing morphology as an equally critical design dimension alongside surface chirality for optimizing chiral nanocarrier performance. Based on this dual-parameter optimization outcome, the lead D-RCO configuration was formulated into the Au RCO@mPEG-SH@siPD-L1 nanoformulation, which achieved robust PD-L1 knockdown and effective proliferation inhibition in H460 lung cancer cells both in vitro and in vivo, without requiring complex targeting ligand modification.

This work provides critical experimental evidence supporting the local antitumor performance of the D-Au RCO nanoplatform, and highlights that further evaluation of its systemic delivery efficacy following intravenous administration will be required to advance future clinical translation. Collectively, these results offer a clear design blueprint for engineering chirality- and morphology-optimized nanocarriers, and open new avenues for the translational application of intrinsically chiral nanomaterials in solid tumor targeted therapy and precision cancer medicine.

## CRediT authorship contribution statement

**Qiusha Li:** Writing – original draft, Project administration, Methodology, Investigation, Conceptualization. **Jinglei Xu:** Methodology, Investigation, Formal analysis, Conceptualization. **En Zhang:** Visualization, Investigation, Formal analysis. **Chen Liu:** Methodology, Investigation, Formal analysis. **Wuyi Liu:** Methodology, Investigation. **Kaichuan Yan:** Supervision, Methodology, Investigation, Formal analysis. **Doudou Xu:** Writing – review & editing, Supervision, Conceptualization. **Jian Wang:** Supervision, Project administration, Funding acquisition.

## Statement of significance

Nanoparticle chirality in non-viral gene delivery has been viewed as a surface ligand property, while intrinsic crystallographic handedness of inorganic cores has remained unexplored. This work orthogonally engineers both intrinsic chiXrality (D/L/DL) and morphology (RCO/Cubes) in Au NPs and decouple their individual and combined contributions to siRNA delivery. It is found that D-handed nanoparticles exhibit significantly faster cellular internalization and superior gene silencing compared with L- and DL-handed counterparts, while morphology further amplifies this chiral advantage, identifying it as an equally critical design dimension. Co-optimization enables anti-PD-L1 siRNA delivery that suppresses lung cancer in vivo without complex targeting ligands, establishing chirality as a fundamental design principle for next-generation nanotherapeutics (see Scheme 1).

**Scheme 1:**
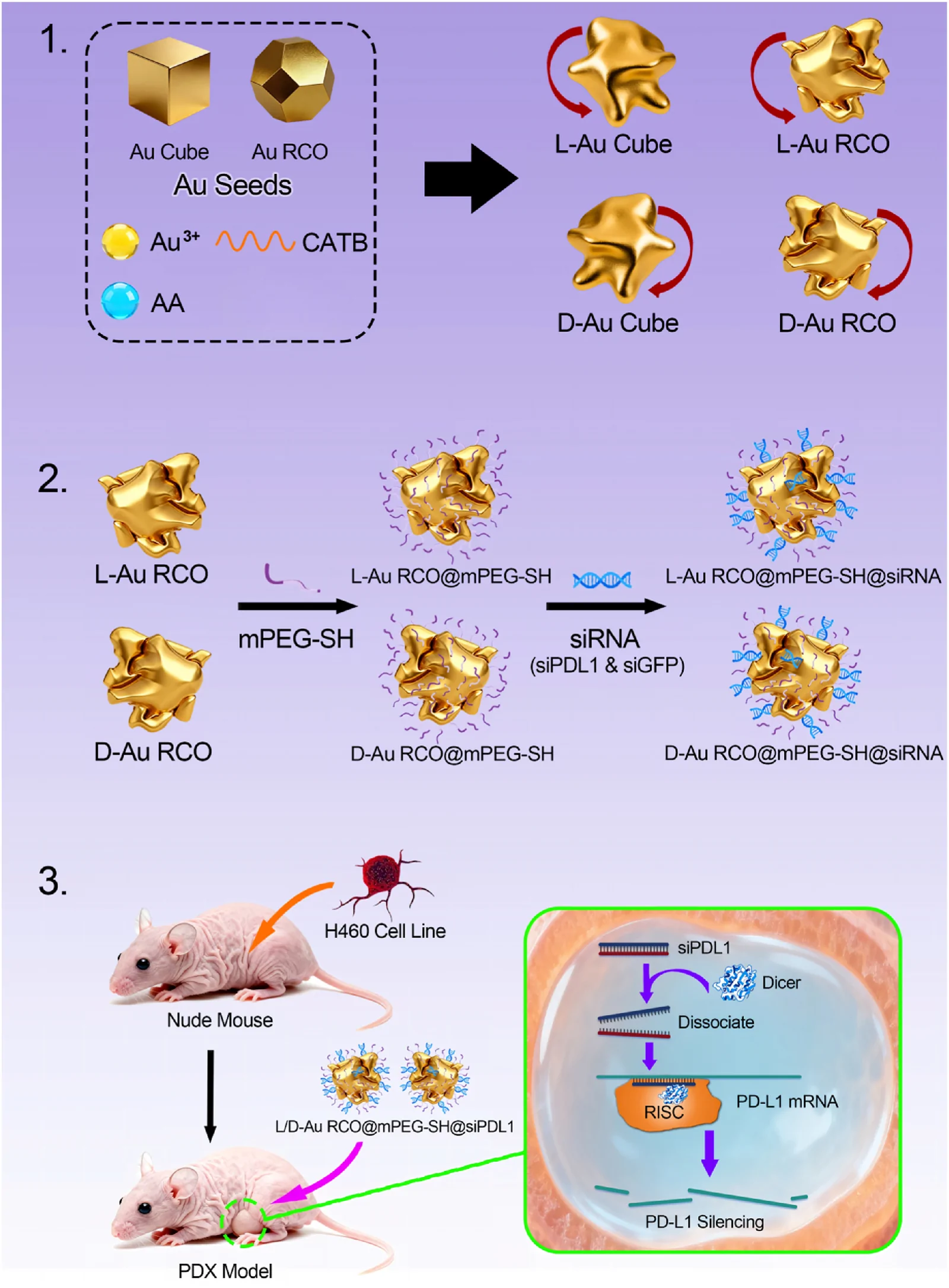
Illustration of intrinsic chiral NPs for chirality-dependent tumor growth inhibition. (1) Preparation of DL-/L-/D-Au NPs. (2) Surface modification of DL-/L-/D-Au NPs and siRNA loading. (3) DL-/L-/D- Au RCO@mPEG-SH@siPD-L1 inhibition proliferation of tumor cells.

## Ethics approval and consent to participate

All animal experiments were approved by Ethic Committee of Southwest University (No. 20260108-04) and were carried out in compliance with the Animal Management Rules of the Ministry of Health of the People's Republic of China (Document NO. 545, 2001).

## Declaration of competing interest

The authors declare that they have no known competing financial interests or personal relationships that could have appeared to influence the work reported in this paper.

## Data Availability

Data will be made available on request.
