# Supplementary material for: Chirality and morphology-dependent siRNA delivery for targeted lung cancer therapy with intrinsically chiral gold nanoparticles
Source: Mater Today Bio. 2026 Oct 9;41:103745. doi: 10.1016/j.mtbio.2026.103745 (PMC13682186; doi:10.1016/j.mtbio.2026.103745)
Supplement: Supplementary file 1 [file mmc1.docx]

**Supporting Information**

**Chirality and morphology-dependent siRNA delivery for targeted lung cancer therapy with intrinsically chiral gold nanoparticles**

Qiusha Li ^a^, Jinglei Xu ^a^, En Zhang ^b^, Chen Liu ^a^, Wuyi Liu ^a^, Kaichuan Yan ^a,*^, Doudou Xu ^c^^,*^ and Jian Wang ^a,*^

*^a^ Key Laboratory of Biomedical Analytics (Southwest University), Chongqing Science and Technology Bureau, College of Pharmaceutical Sciences, Southwest University, Chongqing 400715, P. R. China*

*^b^ Chongqing Key Laboratory of Quality Control and Safety Evaluation of Active Pharmaceutical Ingredients, Chongqing Institute for Food and Drug Control, Chongqing 401121, P. R. China*

*^c^ Chongqing Pharmaceutical Science Technology School, Chongqing 400700, P. R. China*

^*^Corresponding authors.

*E-mail addresses:* yankaichuan@foxmail.com (K. Yan); xdd@cqpsts.cn (D. Xu), wj123456@swu.edu.cn (J. Wang)

**Experimental Section**

**Instruments**

The J810 circular dichroism (CD) spectrometer (JASCO, Japan) was used for scanning CD spectra. The UV-2310 UV-vis spectrophotometer (Techcomp, China) was used to obtain the absorption spectra. The result of X-ray diffraction (XRD) was provided by the D8 ADVANCE X-ray diffractometer (Brooke, Germany) and processed by MDI Jade 6 software (Materials Data, USA). An H1650W high-speed centrifuge (Hunan Xiangyi Company, China) was used to purify sample and an HCJ-6D water bath digital magnetic stirrer (Changzhou Longyue Company, China) was for controlling temperature during the experiments. The flow cytometry data was obtained by BD LSRFortessa Cell Analyzer. All the fluorescence images were captured from High-Content Screening (HCS). All RT-qPCR reactions were performed with a Toptical 96 Real-Time PCR System (Analytikjena, Germany). The Zetasizer Nano (ZEN3600, England, Malvern) was used to obtain the dynamic light scattering spectra (DLS).

**Synthesis of Au seeds**

Single-crystalline gold nanoparticles (Au NPs) were synthesized by the seed-mediated method. Firstly, colloidal Au seeds were prepared, 25 µL of 100 mM chloroauric acid solution was added in 200 mM cetyltrimethylammonium chloride (CTAC). Secondly, 450 µL of pre-cooled 20 mM sodium borohydride solution was added and stirred rapidly for 2 min to allow for a complete oxidation-reduction reaction. Then, the reaction system was placed in a 30°C water bath and left to age for 1 h to obtain uniformly sized and highly crystalline single-crystal gold nanoparticles. Excess CTAC and by-products were removed by centrifugation and washing. Finally, they were dispersed in ultrapure water for storage.

**Synthesis of Single-crystalline Gold Rhombicuboctahedron (RCO) and Gold Cube**

First, two vials of Au NPs growth solutions were prepared with the same composition. For the synthesis of Au RCO: 25 µL of 100 mM chloroauric acid solution was added under the condition of 200 mM CTAC, which was stirred evenly in a water bath at 30 °C. Then, 30 µL of 1 mM KI and 220 µL of 40 mM AA were added in sequence, the total volume of solution in each vial was set to 10.0 mL. As for the growth solution, 10 µL of 10 mM NaBr was added instead of KI, and the amount of L-AA was 90 µL for Au cube, the total solution volume was 10.0 mL. Finally, the synthesized single-crystalline NPs were centrifuged at 8000 rpm for 4 min. After being washed twice with water, they were stored in 1 mL of 1 mM CTAB aqueous solution for subsequent chiral synthesis.

**Supplementary Tables：**

**Table S1**. siRNA sequences used in this work

| siRNA | Sense (5'-3') | Antisense (5'-3') |
| --- | --- | --- |
| siGFP | 5'-GCA CCA UCU UCU UCA AGG AUU-3' | 5'-UCC UUG AAG AAG AUG GUG CUU-SH-3' |
| Cy5-labeled siGFP | 5'-Cy5-GCA CCA UCU UCU UCA AGG AUU-3' | 5'-Cy5-UCC UUG AAG AAG AUG GUG CUU-SH-3' |
| siPD-L1 | 5'-GGC ACA UCC UCC AAA UGA AUU-3' | 5'-UUC AUU UGG AGG AUG UGC CUU-SH-3' |
| B-MM6-siPD-L1 | 5'-GGC ACA UCC UCC AAA UGA AUU -3' | 5'-UUC AUU CCC AGG AUG GAA CUU-SH-3' |

**Table S2**. Gene primer sequences used in RT-qPCR

| Gene | Forward primers | Reverse primers |
| --- | --- | --- |
| PD-L1 | 5'-AGG CCG AAG TCA TCT GGA CAA-3' | 5'-TGA TTC TCA GTG TGC TGG TCA-3' |
| STAT3 | 5'-GCA GCT GAC TAC ACT GGC AGA GA-3' | 5'-ATT GTC CAG CCA GAC CCA GAA-3' |
| Caspase3 | 5'-GAC TCT GGA ATA TCC CTG GAC AAC A-3' | 5'-AGG TTT GCT GCA TCG ACA TCT G-3' |
| GAPDH | 5'-CAG GTT GTC TCC TGC GAC TT-3' | 5'-TAT GGG GGT CTG GGA TGG AA-3' |

**Supplementary Figures**

**Figure S1**. SEM images of (A) Au RCO (B) Au Cube; insets are HRTEM images of single particles. Scale bars: 100 nm (SEM), 20 nm (HRTEM).

**Figure S2.** Structural formula of (A) L-Cys and (B) D-Cys.

**Figure S3**. Elemental mapping of a single (A) DL-Au RCO and (B) L-Au RCO.

**Figure S4**. Size distribution histogram of (A) L-Au RCO, (B)D-Au RCO, (C) DL-Cube, (D) L-Cube and (E)D-Cube.

**Figure S5**. FTIR spectra of (A) mPEG-SH, L-Au RCO and L-Au RCO@mPEG-SH; (B) mPEG-SH, D-Au RCO and D-Au RCO@mPEG-SH.

**Figure S6**. Zeta potential of the (A) L-Au RCO and (B) D-Au RCO after being modified with 0, 10, 30 and 50 nM siGFP, respectively.

**Figure S7**. Zeta potential of DL/L/D-Au Cube before and after modification.

**Figure S8.** MST assays measuring the binding affinity between L/D-Au RCO@mPEG-SH and Cy5-siGFP.

**Figure S9**. CD spectra of the modified DL/L/D-Au RCO and Cube.

**Figure S10.** *g*-factor changes of DL/L/D-Au RCO@mPEG-SH at different time points.

**Figure S11**. SEM images of the modified (A) L- Au RCO and (B) D- Au RCO.

**Figure S12.** EDS analysis of (A) L-Au RCO@mPEG-SH and (B) D-Au RCO@mPEG-SH. n.d., not detected (Br signal below the detection limit under the acquisition conditions used in this experiment). (C) EDS quantitative analysis of the marked point in L-and D-Au RCO@mPEG-SH.

**Figure S13.** Viability of L929 cells after treatment with (A) L-Au RCO@mPEG-SH@siGFP and (B) D-Au RCO@mPEG-SH@siGFP. Error bars represent SEM (n=3).

**Figure S14.** Flow cytometry analysis of the GFP expression after GFP-A549 incubation with DL/L/D-Au Cube@mPEG-SH@siGFP for 24 h.

**Figure S15**. The fluorescence imaging of GFP-A549 cells treated with DL/L/D-Au Cube@mPEG-SH@siGFP for 24h. Scale bar: 50 μm.

B

**Figure S16. (A)** *g*-factor spectra of D (1,2,3)-Au RCO and D-Au Cube@mPEG-SH@siGFP. (B) Fluorescence imaging of GFP-A549 following incubation with D (1,2,3)-Au RCO and D-Au Cube@mPEG-SH@siGFP. Scale bar: 100 μm.

**Figure S17.** (A) GO enrichment analysis of total differentially expressed proteins. (B-D) GO enrichment analysis of differentially expressed proteins in DL-, D-, L-Au RCO group versus the control.

**Figure S18.** Hierarchical clustering heatmap of differentially expressed proteins among the L- and D-Au RCO groups. Relative protein abundance values from biological replicates in each group were Z-score normalized and clustered according to their expression patterns.

**Figure S19**. The cell activity of A549 cells after incubation with DL/L/D-Au RCO@mPEG-SH@siPD-L1. Error bars represent SEM (n=3).

**Figure S20.** Fluorescence imaging of EdU-positive A549 cells following incubation with DL/L/D-Au RCO@mPEG-SH@siPD-L1. Scale bar: 100 μm.

**Figure S21.** Fluorescence imaging of EdU-positive A549 cells after incubation with D-Au RCO@mPEG-SH@siPD-L1, blank vector, scrambled siRNA, or free siPD-L1. Scale bar: 100 μm.

**Figure S22.** The RT-qPCR analysis of (A) *PD-L1 (*B) *STAT3* and (C) *Caspase-3* mRNA levels in A549 cells after treatment with DL/L/D-Au RCO@mPEG-SH@siPD-L1 for 24 h. Error bars represent SEM (n=3).

**Figure S23.** Flow cytometry analysis of H460 cell apoptosis after treatment with PBS, DL/L/D-Au RCO@mPEG-SH@siPD-L1 for 24 h.

**Figure S24.** Immunofluorescence images of A549 cells after treatment with DL/L/D-Au RCO@mPEG-SH@siPD-L1 for 24 h. Scale bar: 100 μm.

**Figure S25.** Quantification of average PD-L1 fluorescence intensity of A549 cells from immunofluorescence images. Error bars represent SEM (n=3).

**Figure S26.** (A) AST, (B) BUN and (C) CREA levels in the serum of mice after different treatments. Error bars represent SEM (n=5).

**Figure S27.** Au contents in major organs of mice from different treatment groups as quantified by ICP-MS: (A) spleen (B) lung and (C) kidney. Error bars represent SEM (n=5).

**Figure S28**. H&E staining of tumor sections in all groups after various treatments. Scale bar: 50 μm.
